# Supplementary material for: CD11c is not required by microglia to convey neuroprotection after prion infection
Source: PLoS One. 2023 Nov 1;18(11):e0293301. doi: 10.1371/journal.pone.0293301 (PMC10619787; doi:10.1371/journal.pone.0293301)
Supplement: S1 Raw images — (PDF) [file pone.0293301.s004.pdf]

CD11c-/- C57BL/6

1 2 3 4 X 5 6 7 8

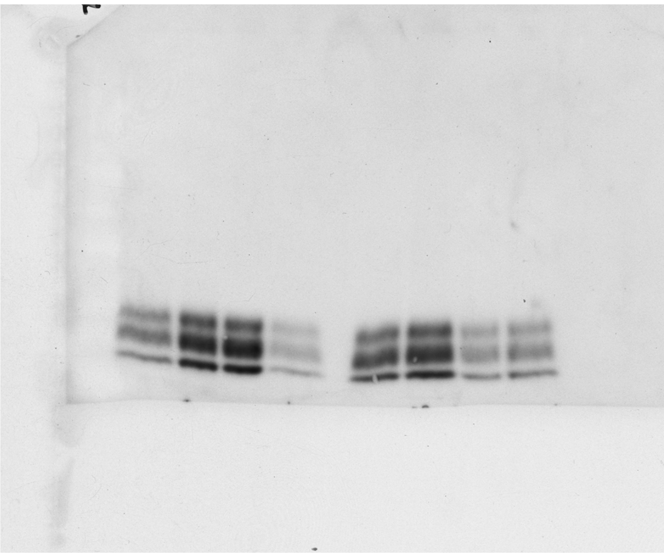

80 dpi Brain Homogenate
Proteinase K treated
Figure 4 A
Uncropped

CD11c-/- C57BL/6

1 2 3 4 X 5 6 7 8

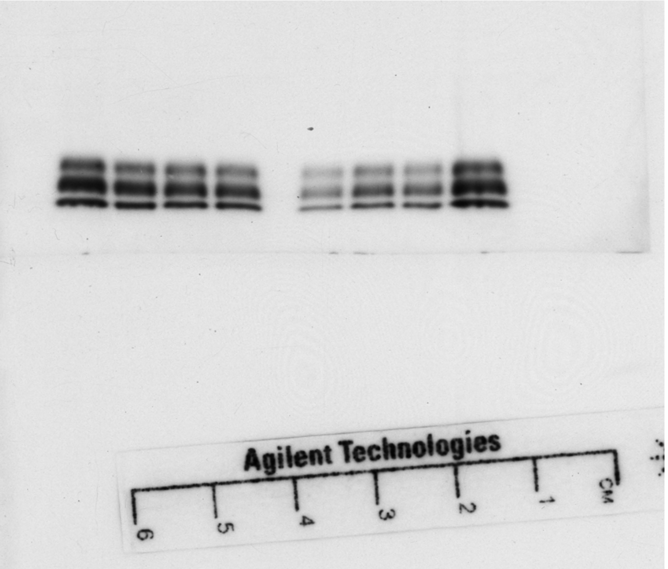

120 dpi Brain Homogenate
Proteinase K treated
Figure 4 B
Uncropped

CD11c-/- C57BL/6

1 2 3 4 5 X 6 7 8 9

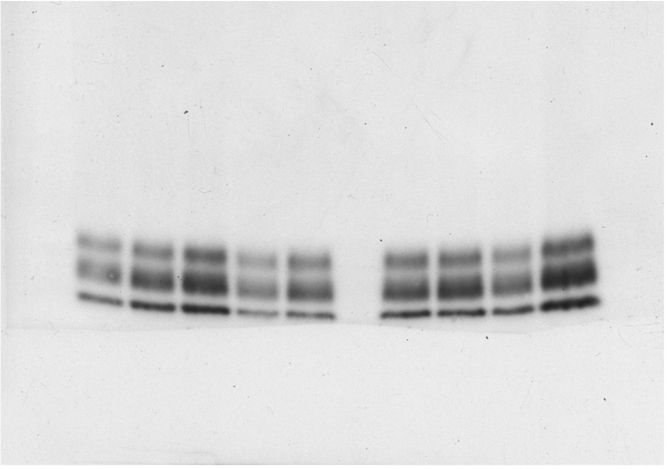

Clinical Brain Homogenate
Proteinase K treated
Figure 4 C
Uncropped
