# Supplementary material for: CD11c is not required by microglia to convey neuroprotection after prion infection
Source: PLoS One. 2023 Nov 1;18(11):e0293301. doi: 10.1371/journal.pone.0293301 (PMC10619787; doi:10.1371/journal.pone.0293301)
Supplement: Attachment — Submitted filename: Response to Reviwer_PONE-D-23-19514.docx [file pone.0293301.s005.docx]

Reviewer #1: The paper by Carroll et al describes prion disease tempo, neuropathogenesis and alterations in brain transcription following experimental inoculation of transgenic mice devoid of CD11c, with the aim of determining whether CD11c-dependant pathways are important in the relative protection offered by reactive microglia. Absence of CD11c was found to be without any notable influence on all features studied. However, the authors identified pathways activated in both wt mice and CD11 ko mice that are of interest, particularly those related to innate immune response and SYK-dependent phagocytosis in the brain.

The results are important as they complement prior work on the role of microglia in prion pathogenesis. The paper is clearly written, technically sound and the conclusions are supported by the data.

One point that would merit further discussion or clarification is the potentially dual role of the activated pathways identified. My understanding is that the authors suggest that the SYK-dependent pathways are potentially “protective” because associated with phagocytosis and potential prion clearance. On the other hand, such inflammatory-related pathways may be detrimental. For example, Combs et al (PMID:9920656) linked neurotoxicity to activation of Lyn/Syk-dependent pathways, following stimulation of primary microglial cell by Ab and PrP fibrils. Both kinases are activated in the present manuscript, raising the question of their real role in prion neuropathogenesis.

**This is an excellent point by the reviewer. We have expanded the Discussion section by adding 3 paragraphs to cover the involvement of Lyn/Syk in prion and other neurodegenerative diseases. This should help to bridge our findings with RML prion infection of mice and these other relevant neurological disorders. We believe this is a great suggestion by the reviewer and has improved the manuscript.**

What is the genetic background of CD11c ko mice? C57BL/6? This information is important to ensure that comparison of transcriptomes can be made between wt and CD11c ko mice.

**We apologize for this oversight on our part. The CD11c -/- mice that we obtained from Drs. Wu and Ballantyne were originally backcrossed onto the C57BL/6 strain for at least seven generations. We have added this important detail in the Materials and Methods section under the subcategory “Mice.”**
